# Quantitative Evaluation of Peripapillary Structure and Microcirculation in Children and Adolescents With Myopia

**DOI:** 10.1155/joph/5746498

**Published:** 2026-08-16

**Authors:** Shaojin Zhu, Yunyi Wang, Mengqian Cai, Chi Xie, Yan Fang

**Affiliations:** ^1^ The First Affiliated Hospital of Anhui University of Science and Technology, Huainan 232000, Anhui, China; ^2^ Eye Institute of Anhui University of Science and Technology, Huainan 232000, Anhui, China

**Keywords:** axial length, myopia, peripapillary microcirculation, peripapillary structure, swept-source optical coherence tomography angiography

## Abstract

**Purpose:**

To characterize alterations in peripapillary structural and microvascular features in children and adolescents with myopia across different stages of severity using swept‐source optical coherence tomography angiography (SS‐OCTA).

**Methods:**

This cross‐sectional study was conducted with the enrollment of 106 eyes from 54 children. Three groups of Group A (AL ≤ 24 mm, 34 eyes), Group B (24 < AL < 25 mm, 37 eyes), and Group C (AL ≥ 2 5 mm, 35 eyes) were established based on the axial length (AL) of the included participants. All participants completed a standardized ophthalmic examination protocol, including best‐corrected visual acuity (BCVA), autorefraction, AL and SS‐OCTA. The specific parameters were the peripapillary retinal thickness (pRT), peripapillary choroidal thickness (pCT), peripapillary superficial vascular complex density (pSVD), peripapillary deep vascular complex density (pDVD), peripapillary choroidal vessel volume (pCVV) and peripapillary choroidal vascular index (pCVI). Data were analyzed using one‐way analysis of variance and Pearson correlation analysis.

**Results:**

There were significant differences in pRT within the NS, NI, IT, TS, and ST regions with increasing AL (*p* < 0.05). The value of pCT decreased in the IT, TI, TS, and ST regions, while pDVD declined in the NS, IT, TI, TS, and ST regions, and pCVV decreased in the NS, NI, IN, IT, TI, and TS regions. Conversely, pSVD increased in the NS, NI, IT, TI, TS, and ST regions (all *p* < 0.05). Furthermore, significant positive correlations were found between pRT and pSVD, pDVD and pCT, pCVV and pCT, pCVV and pDVD, as well as pCVI and pCVV (*R* = 0.346, 0.297, 0.946, 0.298, and 0.361, all *p* < 0.05, respectively). Meanwhile, significant negative correlations were observed between AL and pCT, AL and pDVD, AL and pCVV, pRT and pDVD, pRT and pCVI, pSVD and pDVD, as well as pSVD and pCVV (*R* = −0.304, −0.236, −0.288, −0.402, −0.195, −0.332, and −0.203, all *p* < 0.05, respectively).

**Conclusion:**

The pRT shows a significant thinning trend when AL exceeds 25 mm. AL has negative correlations with pCT and pDVD, but positive correlations with pSVD in the IT, TI, ST, and TS regions. These findings highlight the potential of peripapillary structural parameters for monitoring myopia progression.

## 1. Introduction

Myopia in children and adolescents has emerged as a pressing global public health concern. The trend of myopia prevalence shifting toward younger ages may seriously compromise the visual health of children and adolescents. In view of the rapidly rising prevalence of myopia and high myopia globally, it is projected that 49.8% of the global population (4758 billion) will be myopic, and 9.8% (938 million) will have high myopia by 2050 [1]. In China, the prevalence of myopia reached 52.7% among children and adolescents in 2020, with a trend toward earlier onset, and it is estimated to increase to 61.8% in those aged 6–18 years by 2030 [2]. In Chinese children and adolescents, the overall myopia prevalence was 36.6%: 22.0% at 5–9 years, 45.4% at 10–14 years, and 67.2% at 15–19 years. The overall high myopia prevalence was 5.3%, with 1.1%, 3.0%, and 9.5% in the same age groups, respectively [3]. Given the associated risk of irreversible visual impairment, the high prevalence of myopia imposes a substantial economic and social burden. Axial length (AL) is a key indicator of myopia progression, and there may be distinct pathological alterations of the structural and microvascular characteristics of the fundus as AL increases, particularly in highly myopic eyes [4]. AL exceeding 26 mm has been demonstrated to be associated with a significantly elevated risk of visual impairment, such as retinal detachment, choroidal neovascularisation, macular pucker, and posterior staphylomas. [5]. Swept‐source optical coherence tomography angiography (SS‐OCTA) is a noninvasive fundus imaging technique that produces high‐resolution images. SS‐OCTA in myopia allows for a faster, wider‐scope, and more comprehensive assessment of the structural and microvascular features of the retina and choroid, exhibiting significant clinical and research value [6, 7]. Critically, patients with myopia are usually observed with significant alterations in retinal and choroidal structure and microcirculation. Increase in AL occurs concurrently with the decrease of peripapillary vascular density (pVD), particularly in the inferotemporal (IT), temporoinferior (TI), and temporosuperior (TS) regions. Notably, pVD in highly myopic patients displays no significant association with age or spherical equivalent (SE) [8]. Additionally, subjects with early‐onset myopia may present with progressive choroidal thinning, while the severity of thinning reveals no evident correlation with disease duration [9]. In myopic traction maculopathy, peripapillary choroidal thickness (pCT) has a significant negative correlation with microcirculation, highlighting distinct pathological mechanisms underlying this vision‐threatening complication. Additionally, peripapillary choroidal microvascular alterations showed positive correlation with SE and negative correlation with AL in adolescents and young adults with high myopia [10].

Accordingly, this study employed SS‐OCTA for quantitative tissue analysis to further elucidate the distinct structural and microvascular changes in peripapillary regions of myopic children and adolescents. This study focused on investigating the clinical manifestations and pathological mechanisms underlying optic disc changes during myopia progression and hence establishing evidence‐based reference values. These findings may guide targeted clinical strategies for myopia prevention and control, thereby addressing the substantial public health burden posed by this highly prevalent refractive disorder.

## 2. Materials and Methods

### 2.1. Ethical Approval

In accordance with the principles of the Declaration of Helsinki, this study was conducted with official approval issued by the institutional review board and Ethical Committee of the First Affiliated Hospital of Anhui University of Science and Technology (No. 2022‐KY‐017‐001). Informed consent was obtained from the guardian of each subject.

### 2.2. Subjects

This study was initiated with the recruitment of children and adolescents, including both myopic participants and nonmyopic controls, from the First Affiliated Hospital of Anhui University of Science and Technology between July and August 2023. Inclusion criteria: (1) age of 6–17 years; (2) best‐corrected visual acuity (BCVA) ≥ 1.0; (3) intraocular pressure (IOP) between 10 and 21 mmHg; (4) SE between −10.00D and +1.50D, with cylindrical power ≤ 2.00D in both eyes; and (5) signal strength index of OCTA ≥ 6; Exclusion criteria: (1) patients whose guardians refused to participate in the test; (2) patients with systemic diseases such as hypertension, diabetes and systemic connective tissue disorder; (3) patients with pathologic myopic fundus changes, previous vitreoretinal surgery, primary or secondary glaucoma, any media opacity affecting fundus imaging, and poor imaging quality of OCTA; and (4) patients with inability to cooperate with the examination.

### 2.3. Methods

**FIGURE 1:**
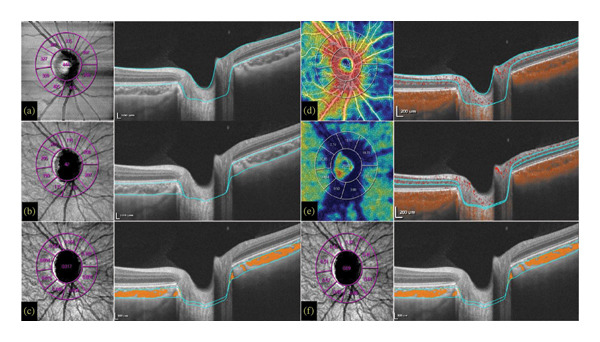
The peripapillary structure and microcirculation parameters. (a) Peripapillary retinal thickness (pRT), (b) peripapillary choroidal thickness (pCT), (c) peripapillary choroidal vessel volume (pCVV), (d) peripapillary superficial vascular complex density (pSVD), (e) peripapillary deep vascular complex density (pDVD), and (f) peripapillary choroidal vascular index (pCVI).

This was a cross‐sectional study. Three groups were constructed based on the status of AL, including Group A (AL ≤ 24 mm, 34 eyes), Group B (24 < AL < 25 mm, 37 eyes), and Group C (AL ≥ 25 mm, 35 eyes). A standardized ophthalmic examination protocol was established in this study, including slit‐lamp biomicroscopy, BCVA, IOP measurement (Topcon, Japan), autorefraction (Topcon Corporation, Japan), AL measurement using IOL Master 700 (Carl Zeiss Meditec, Germany), and SS‐OCTA using VG200S VG200S (SVision Imaging, Henan, China). The optic nerve head angiography was performed using 6 × 6 mm scans. The peripapillary region was defined as a ring area centered on the optic disc, with inner and outer diameters of 2 and 4 mm, respectively. In the application of the SS‐OCTA system, the following parameters were automatically measured, including the peripapillary retinal thickness (pRT), pCT, peripapillary superficial vascular complex density (pSVD), peripapillary deep vascular complex density (pDVD), peripapillary choroidal vessel volume (pCVV), and peripapillary choroidal vascular index (pCVI). As illustrated in Figure 1, the peripapillary region was divided into 8 sectors (superonasal, SN; nasosuperior, NS; nasoinferior, NI; inferonasal, IN; inferotemporal, IT; temporoinferior, TI; temporosuperior, TS; superotemporal, ST) according to the Garway–Heath method. All these peripapillary parameters were assessed by two experienced retinal specialists independently. Specific participants would be excluded if a diagnostic consensus could not be reached.

### 2.4. Statistical Analysis

Statistical analyses were performed using SPSS 22.0 (SPSS Inc., Chicago, IL, USA). Data are presented as mean ± standard deviation (SD). Normality of data distribution was evaluated using the Shapiro–Wilk test. Intergroup comparisons were performed by one‐way analysis of variance. Correlations between variables were determined by employing the Pearson correlation analysis. A *p* value < 0.05 was considered statistically significant. The figure was drawn by https://www.chiplot.online.

## 3. Results

### 3.1. Demographic Data

This study was conducted with the inclusion of 54 individuals (106 eyes), including 29 boys and 25 girls. There existed no significant differences in IOP among the three groups (*p* > 0.05), whereas significant differences were found in AL, age, sex distribution, and SE (all *p* < 0.05). Demographic and clinical characteristics of each group are detailed in Table 1.

**TABLE 1:** Clinical characteristics of the subjects.

Groups	Age (y)	Sex (M/F)	IOP (mmHg)	AL (mm)	SE (D)
Group A	8.79 ± 2.63	12/7	16.66 ± 2.77	23.42 ± 0.44	−0.81 ± 1.33
Group B	10.41 ± 2.68	9/14	17.46 ± 2.78	24.44 ± 0.28	−2.04 ± 1.54
Group C	12.86 ± 2.89	12/8	16.87 ± 3.01	26.03 ± 0.67	−5.45 ± 1.72
*p* value	< 0.001^∗^	< 0.001^∗^	0.478	< 0.001^∗^	< 0.001^∗^

*Note:* Data are presented as mean ± standard deviation unless otherwise indicated. Sex data are shown as number of males/females. D, dioptre; IOP, intraocular pressure; M, male; F, female.

Abbreviations: AL, axial length; SE, spherical equivalent.

^∗^ *p* < 0.05: statistically significant.

### 3.2. Alterations in Peripapillary Structure and Microcirculation Parameters

**FIGURE 2:**
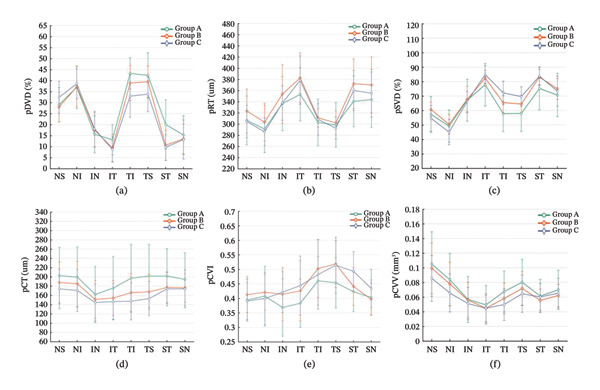
Line charts showing the differences of peripapillary parameters among the three groups. (a) Line chart of pRT, (b) line chart of pDVD, (c) line chart of pSVD, (d) line chart of pCT, (e) line chart of pCVI, and (f) line chart of pCVV.

With increasing AL, multiple peripapillary parameters changed progressively, with region‐specific patterns. Detailed regional data are presented in Table 2 and Figure 2, with key findings summarized as follows. Mean pRT increased with longer AL in Groups A and B (AL ≤ 25 mm) but declined in Group C (AL > 25 mm), suggesting an inflection point at approximately 25 mm. Within the NS, NI, IT, TS, and ST regions, there were significant differences among the three groups (all *p* < 0.05). Both pCT and pCVV decreased progressively across groups. The value of pCT was the thickest in the NS region but the thinnest in the IN region. Furthermore, pCT reduced significantly in the IT, TI, TS, and ST regions (all *p* < 0.05), while pCVV decreased significantly in the NI, TI, and TS regions (all *p* < 0.05). In contrast to other microvascular parameters, pSVD increased with longer AL in the IT, TI, TS, and ST regions (all *p* < 0.05). However, pSVD was highest in Group B and the lowest in Group C in the NS and NI regions. Meanwhile, the pDVD values decreased progressively with increasing AL in the IT, TI, TS, and ST regions (all *p* < 0.05). Conversely, pDVD was the highest in Group C within the NS region. The pCVI showed significant regional variation, with decreases in the IN, IT, TS, ST, and SN regions (all *p* < 0.05). In addition, Group A exhibited the lowest pCVI across most temporal regions.

**TABLE 2:** Comparison of peripapillary parameters across regions among the three groups.

Parameters	NS	NI	IN	IT	TI	TS	ST	SN
pRT (um)								
Group A	312.91 ± 23.34	297.11 ± 23.87	343.63 ± 26.73	360.84 ± 21.48	309.21 ± 20.45	304.54 ± 17.93	75.25 ± 14.83	351.09 ± 26.98
Group B	323.33 ± 39.46	303.46 ± 33.86	353.79 ± 52.47	382.57 ± 44.86	310.97 ± 16.64	302.34 ± 21.3	83.07 ± 5.31	369.95 ± 50.26
Group C	304.62 ± 28.91	286.05 ± 24.58	337.7 ± 30.73	378.52 ± 43.25	308.24 ± 28.06	292.78 ± 19.84	83.68 ± 6.44	355.29 ± 42.84
*F*	3.19	3.55	1.59	3.17	0.14	3.48	4.07	2.05
*p* value	0.045^∗^	0.032^∗^	0.209	0.046^∗^	0.868	0.034^∗^	0.02^∗^	0.135
pCT (um)								
Group A	202.64 ± 61.09	199.35 ± 64.92	161.93 ± 60.51	175.62 ± 68.3	197.04 ± 73.16	201.68 ± 68.22	201.58 ± 59.44	194.52 ± 57.05
Group B	187.8 ± 44.25	184.93 ± 48.78	151.18 ± 40.87	154.24 ± 37.69	165.81 ± 41.5	167.6 ± 38.34	176.9 ± 32.75	176.14 ± 30.79
Group C	173.93 ± 42.94	170.6 ± 45.59	144.51 ± 40.21	146.63 ± 38.84	147.32 ± 40.15	153.12 ± 36.96	173.87 ± 36.78	174.09 ± 41
*F*	2.858	2.486	1.162	3.128	7.637	8.715	4.087	2.268
*p* value	0.062	0.088	0.317	0.048^∗^	< 0.001^∗^	< 0.001^∗^	0.020^∗^	0.109
pSVD (%)								
Group A	57.31 ± 12.38	49.22 ± 11.1	67.1 ± 14.43	77.78 ± 14.71	57.8 ± 12.4	57.93 ± 12.46	75.25 ± 14.83	70.66 ± 14.95
Group B	60.64 ± 5.15	50.38 ± 6.9	67.64 ± 9.07	82.39 ± 5.37	65.42 ± 7.31	64.48 ± 5.86	83.07 ± 5.31	74.93 ± 7.18
Group C	54.24 ± 8.69	44.99 ± 8.43	65.74 ± 6.79	84.93 ± 6.29	72.54 ± 8.14	69.84 ± 6.79	83.68 ± 6.44	73.4 ± 10.48
*F*	4.423	3.589	0.309	4.891	20.833	15.948	8.129	1.308
*p* value	0.014^∗^	0.031^∗^	0.735	0.009^∗^	< 0.001^∗^	< 0.001^∗^	< 0.001^∗^	0.275
pDVD (%)								
Group A	29.03 ± 7.79	36.99 ± 9.7	15.62 ± 8.39	13.12 ± 6.88	43.27 ± 7.08	42.39 ± 10.23	20.07 ± 11.17	15.28 ± 8.78
Group B	28 ± 6.71	37.21 ± 9.15	17.55 ± 7.97	9.4 ± 6.16	38.88 ± 7.94	39.49 ± 7.17	10.66 ± 6.83	13.49 ± 6.8
Group C	32.84 ± 7.26	38.73 ± 6.77	18.61 ± 7.81	8.98 ± 5.77	32.98 ± 9.45	33.99 ± 7.69	10.01 ± 5.88	13.35 ± 9.13
*F*	4.387	0.42	1.221	4.548	13.634	8.897	16.222	0.589
*p* value	0.015^∗^	0.658	0.299	0.013^∗^	< 0.001^∗^	< 0.001^∗^	< 0.001^∗^	0.557
pCVV (mm^3^)								
Group A	0.11 ± 0.05	0.08 ± 0.04	0.06 ± 0.03	0.05 ± 0.03	0.07 ± 0.03	0.08 ± 0.03	0.06 ± 0.02	0.07 ± 0.03
Group B	0.1 ± 0.03	0.08 ± 0.03	0.06 ± 0.02	0.04 ± 0.02	0.06 ± 0.02	0.07 ± 0.02	0.06 ± 0.02	0.06 ± 0.02
Group C	0.08 ± 0.03	0.07 ± 0.03	0.05 ± 0.02	0.04 ± 0.02	0.05 ± 0.02	0.06 ± 0.03	0.06 ± 0.02	0.07 ± 0.02
*F*	2.885	3.264	0.422	0.656	5.602	3.521	1.231	1.401
*p* value	0.060	0.042^∗^	0.657	0.521	< 0.001^∗^	0.033^∗^	0.296^∗^	0.251
pCVI								
Group A	0.39 ± 0.07	0.41 ± 0.1	0.37 ± 0.1	0.38 ± 0.08	0.46 ± 0.08	0.45 ± 0.09	0.42 ± 0.07	0.4 ± 0.06
Group B	0.41 ± 0.06	0.42 ± 0.06	0.41 ± 0.09	0.43 ± 0.1	0.5 ± 0.1	0.52 ± 0.08	0.44 ± 0.07	0.4 ± 0.06
Group C	0.39 ± 0.06	0.4 ± 0.09	0.42 ± 0.07	0.44 ± 0.1	0.48 ± 0.12	0.51 ± 0.1	0.49 ± 0.07	0.43 ± 0.07
*F*	1.159	0.573	3.691	3.55	1.526	5.822	10.353	3.852
*p* value	0.318	0.566	0.028^∗^	0.032^∗^	0.222	< 0.001^∗^	< 0.001^∗^	0.024^∗^

*Note:* Data are presented as mean ± standard deviation unless otherwise indicated. pSVD, peripapillary superficial vascular complex density; pDVD, peripapillary deep vascular complex density; SN, superonasal; NS, nasosuperior; NI, nasoinferior; IN, inferonasal; IT, inferotemporal; TI, temporoinferior; TS, temporosuperior; ST, superotemporal.

Abbreviations: pCT, peripapillary choroidal thickness; pCVI, peripapillary choroidal vascular index; pCVV, peripapillary choroidal vessel volume; pRT, peripapillary retinal thickness.

^∗^ *p* < 0.05: statistically significant.

### 3.3. Correlation Analysis Between AL and Peripapillary Parameters

Table 3 describes the specific relationship between AL and peripapillary parameters based on the Pearson correlation analysis. The correlation between AL and pRT was significantly positive in IT region (*R* = 0.231, *p* < 0.05) and negative in TS region (*R* = −0.218, *p* < 0.05). AL was significantly negatively correlated with pCT in NS, NI, IT, TI, TS, ST, and SN regions (*R* = −0.231, −0.197, −0.282, −0.432, −0.432, −0.291, and −0.210, all *p* < 0.05, respectively). At the same time, pSVD exhibited significant positive correlations with AL in the IT, TI, TS, ST regions (*R* = 0.303, 0.552, 0.531, and 0.319, all *p* < 0.05, respectively), but a significant negative correlation in the NI region (*R* = −0.276, *p* < 0.05). AL was significantly positively correlated with pDVD in the NS and IN regions (*R* = 0.227, and 0.213, *p* < 0.05, respectively), but with significant negative correlations in the IT, TI, TS, and ST region (*R* = −0.199, −0.503, −0.432, −0.416, all *p* < 0.05, respectively). Besides, significant negative correlations between AL and pCVV were observed in the NS, NI, TI, and TS regions (*R* = −0.268, −0.278, −0.419, and −0.364, all *p* < 0.05, respectively). A significant positive correlation of pCVI with AL was found in the ST region only (*R* = 0.318, *p* < 0.05).

**TABLE 3:** The relationship between AL and peripapillary parameters in different regions among the three groups.

	AL‐pRT		AL‐pCT		AL‐pSVD		AL‐pDVD		AL‐pCVV		AL‐pCVI	
	*R*	*p*	*R*	*p*	*R*	*p*	*R*	*p*	*R*	*p*	*R*	*p*
NS	−0.108	0.272	−0.231	0.017^∗^ ^b^	−0.185	0.058	0.227	0.020^∗^ ^a^	−0.268	< 0.001^∗^ ^b^	−0.111	0.258
NI	−0.153	0.117	−0.197	0.043^∗^ ^b^	−0.276	< 0.001^∗^ ^b^	0.074	0.453	−0.278	< 0.001^∗^ ^b^	−0.132	0.177
IN	−0.064	0.514	−0.156	0.11	−0.123	0.209	0.213	0.028^∗^ ^a^	−0.129	0.188	0.141	0.149
IT	0.231	0.017^∗^ ^a^	−0.282	< 0.001^∗^ ^b^	0.303	< 0.001^∗^ ^a^	−0.199	0.041^∗^ ^b^	−0.175	0.072	0.157	0.108
TI	−0.023	0.811	−0.423	< 0.001^∗^ ^b^	0.552	< 0.001^∗^ ^a^	−0.503	< 0.001^∗^ ^b^	−0.419	< 0.001^∗^ ^b^	−0.059	0.549
TS	−0.218	0.025^∗^ ^b^	−0.432	< 0.001^∗^ ^b^	0.531	< 0.001^∗^ ^a^	−0.432	< 0.001^∗^ ^b^	−0.364	< 0.001^∗^ ^b^	0.114	0.247
ST	0.136	0.165	−0.291	< 0.001^∗^ ^b^	0.310	< 0.001^∗^ ^a^	−0.416	< 0.001^∗^ ^b^	−0.119	0.225	0.318	< 0.001^∗^ ^a^
SN	0.058	0.556	−0.210	0.031^∗^ ^b^	0.111	0.255	−0.119	0.225	−0.141	0.148	0.178	0.068

*Note:* Values represent Pearson correlation coefficients (*R*). pSVD, peripapillary superficial vascular complex density; pDVD, peripapillary deep vascular complex density; SN, superonasal; NS, nasosuperior; NI, nasoinferior; IN, inferonasal; IT, inferotemporal; TI, temporoinferior; TS, temporosuperior; ST, superotemporal. *p* ^∗a^: Significant positive correlation; *p* ^∗b^: Significant positive correlation.

Abbreviations: pCT, peripapillary choroidal thickness; pCVI, peripapillary choroidal vascular index; pCVV, peripapillary choroidal vessel volume; pRT, peripapillary retinal thickness.

^∗^ *p* < 0.05: statistically significant.

**FIGURE 3:**
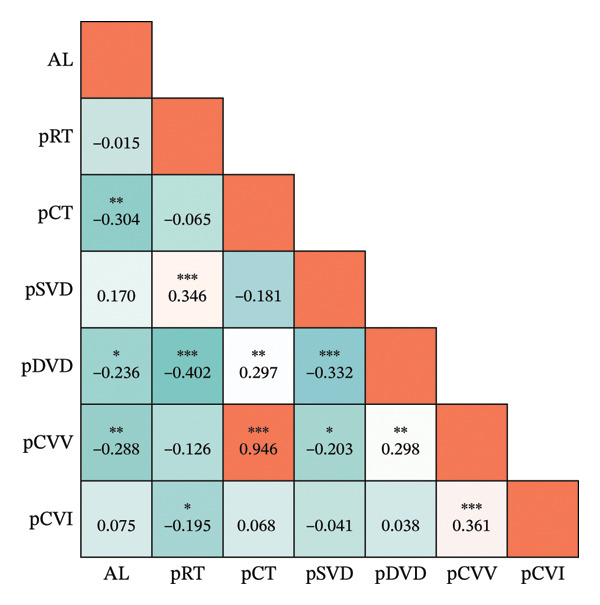
Correlation heatmap of peripapillary parameters.

As depicted in Figure 3, this study continued to clarify the specific relationships between mean peripapillary parameters and AL using Pearson correlation analysis. Consequently, significant positive correlations were noticed between pRT and pSVD, pDVD and pCT, pCVV and pCT, pCVV and pDVD, as well as pCVI and pCVV (*R* = 0.346, 0.297, 0.946, 0.298, and 0.361, all *p* < 0.05, respectively). Simultaneously, significant negative correlations were observed between AL and pCT, AL and pDVD, AL and pCVV, pRT and pDVD, pRT and pCVI, pSVD and pDVD, as well as pSVD and pCVV (*R* = −0.304, −0.236, −0.288, −0.402, −0.195, −0.332, and −0.203, all *p* < 0.05, respectively).

## 4. Discussion

This study analyzed the correlations of peripapillary parameters with AL based on the measurement of specific indices including pRT, pCT, pSVD, pDVD, pCVV, and pCVI. Specifically, our data observed a nonuniform pattern of changes in pRT with increasing AL. AL exhibited a significant positive correlation with pRT in the IT region and a negative correlation in the TS region. Notably, an inflection point at approximately 25 mm was identified given that pRT increased with AL in Groups A and B (AL ≤ 25 mm) but decreased in Group C (AL > 25 mm). This inflection may serve as a predictive biomarker for the transition to high myopia. As reported previously, foveal thickness correlates positively with AL, likely attributable to tangential traction from the internal limiting membrane or posterior vitreous cortex [11–14]. Mishra et al. documented a decrease in peripapillary retinal nerve fiber layer thickness by 1.03 μm per 1 mm increase in AL [15]. The discrepancy may stem from differences in age groups, as our cohort consisted of children and adolescents with developing peripapillary structures potentially.

Structural and functional alterations in the choroid are pivotal in the pathogenesis of numerous ocular diseases. As evidenced by a growing body of research in myopia pathogenesis, choroidal dysfunction can significantly drive axial elongation and subsequent development of myopic maculopathy. In our observation of choroidal structure, both pCT and pCVV exhibited significant regional variations and progressive decrease with increasing AL. Among these, pCT was the thickest in the NS region and the thinnest in the IN region, while pCVV paralleled this topographic pattern. Consistently, Cui et al. [16] and Wu et al. [17] both mentioned the thickest peripapillary choroid in the NS region and the thinnest in the IT regions in myopic children and young adults. The proposed interstudy consistency may suggests a characteristic regional pattern in pCT distribution stemming possibly from the embryonic development of the optic fissure, which closes inferiorly in utero and potentially predisposes the inferonasal quadrant to relative thinning [18]. Notably, our identification of a significant positive correlation of pCT with pCVV may imply that choroidal thinning in myopia is primarily driven by the reduction in choroidal vessel volume. Similarly, Song et al. [19] proposed decreased pCT with axial elongation, and innovatively, our study extended their work through the quantification of the specific vascular contribution to this thinning. Reduction in choroidal vascularity may increase vascular resistance and reduce blood flow, rendering the choroid more susceptible to hypoxia and mechanical deformation, in turn facilitating further axial elongation.

As for choroidal vascular health, pCVI correlated positively with pCVV but negatively with pRT, and it also displayed a positive correlation with AL in the ST region. CVI defines the ratio of choroidal luminal area to total choroidal area quantitatively, functioning as a robust, independent biomarker for assessing choroidal vascular status, independent of factors such as AL, IOP, and age in healthy populations [20]. Unlike the view that macular CVI remains independent of AL reported previously [21, 22], our findings suggest that pCVI may be more sensitive to axial elongation, which may be explained by the unique biomechanical and vascular microenvironment of the optic disc region. In the future, there is a need to clarify the mechanism underlying the dynamic change of pCVI across the entire myopia severity spectrum and its potential role as an early predictive marker of choroidal dysfunction.

Regarding peripapillary microcirculation, a dichotomy was noticed in the response pattern of the superficial and deep vascular complexes to progressive axial elongation. As AL increases progressively, pSVD elevated significantly in the IT, TI, TS, and ST regions, while pDVD decreased concurrently in these same regions. Therefore, the deep vascular complex may be more vulnerable to mechanical stretch than the superficial network. Moreover, pDVD correlated positively with pCT, supporting the hypothesis of concurrent occurrence of choroidal thinning and deep vascular loss. AL and pVD have been found to be negatively correlated in prior studies using nonstratified pVD [23, 24]. Noticeably, our study utilized separate analysis of the superficial and deep vascular complexes, providing a more granular and mechanistic understanding of how these distinct vascular layers respond differently to progressive axial elongation in myopia. The arterial circle of Zinn–Haller, which supplies the optic nerve head, may move farther from the optic disc border with the increase of AL [25], which may compromise the deep perfusion eventually. Meanwhile, highly myopic eyes exhibited significantly lower pDVD and thinner pCT [26]. Wang et al. [27] found the lowest pCVI and pVD in highly myopic eyes compared to healthy eyes, suggesting reduced peripapillary perfusion. As a result, these microvascular alterations may heighten the susceptibility of the optic nerve to glaucomatous damage in high myopia, particularly in the presence of elevated IOP.

In conclusion, SS‐OCTA can yield quantitative and accurate data on peripapillary structural and microvascular changes in myopic children and adolescents. pRT undergoes an inflection when AL exceeds 25 mm, which may serve as a key indicator of progression to high myopia. pDVD is the first to be affected with increasing AL, and AL is negatively correlated with pCT and pDVD while positively correlated with pSVD in the IT, TI, ST, and TS regions. These changes provide important insights into the pathogenesis of myopia, all of which can be regarded as valuable indicators for monitoring disease progression.

It should be acknowledged that the present study is subject to several limitations. First, due to the relatively smaller sample size, our findings should be validated by longitudinal studies with larger cohorts. Second, AL may affect the lateral magnification of OCTA images, potentially influencing quantitative measurements. Third, this study focused exclusively on the effect of AL on peripapillary parameters, without further analysis of the independent contribution of SE. Altogether, these limitations remain to be addressed by future studies with more well‐established experimental designs.

## Funding

This study was supported by the Anhui Health Research Program, AHWJ2022b109 and The Tenth Batch of “50 Stars of Science and Technology” Innovative Projects in Huainan.

## Conflicts of Interest

The authors declare no conflicts of interest.

## Data Availability

The data that support the findings of this study are available from the corresponding author upon reasonable request.
